# Types and severity of medication errors in Iran; a review of the current literature

**DOI:** 10.1186/2008-2231-21-49

**Published:** 2013-06-20

**Authors:** Ava Mansouri, Alireza Ahmadvand, Molouk Hadjibabaie, Mona Kargar, Mohammadreza Javadi, Kheirollah Gholami

**Affiliations:** 1 Research Center for Rational Use of Drugs, Tehran University of Medical Sciences, Tehran, Iran; 2 Department of Epidemiology and Biostatistics, School of Public Health, Tehran University of Medical Sciences, Tehran, Iran; 3 Faculty of Pharmacy, and Research Center for Rational Use of Drugs, Tehran University of Medical Sciences, Tehran, Iran; 4 4th floor, No. 92, Karimkhane Zand Street, 1584775311, Tehran, Iran

**Keywords:** Medication errors, Drug use errors, Patient safety, Prescribing, Transcribing, Drug administration, Dispensing, Pharmacists, Nursing staff, Iran

## Abstract

Medication error (ME) is the most common single preventable cause of adverse drug events which negatively affects patient safety. ME prevalence is a valuable safety indicator in healthcare system. Inadequate studies on ME, shortage of high-quality studies and wide variations in estimations from developing countries including Iran, decreases the reliability of ME evaluations. In order to clarify the status of MEs, we aimed to review current available literature on this subject from Iran. We searched Scopus, Web of Science, PubMed, CINAHL, EBSCOHOST and also Persian databases (IranMedex, and SID) up to October 2012 to find studies on adults and children about prescription, transcription, dispensing, and administration errors. Two authors independently selected and one of them reviewed and extracted data for types, definitions and severity of MEs. The results were classified based on different stages of drug delivery process. Eighteen articles (11 Persian and 7 English) were included in our review. All study designs were cross-sectional and conducted in hospital settings. Nursing staff and students were the most frequent populations under observation (12 studies; 66.7%). Most of studies did not report the overall frequency of MEs aside from ME types. Most of studies (15; 83.3%) reported prevalence of administration errors between 14.3%-70.0%. Prescribing error prevalence ranged from 29.8%-47.8%. The prevalence of dispensing and transcribing errors were from 11.3%-33.6% and 10.0%-51.8% respectively. We did not find any follow up or repeated studies. Only three studies reported findings on severity of MEs. The most reported types of and the highest percentages for any type of ME in Iran were administration errors. Studying ME in Iran is a new area considering the duration and number of publications. Wide ranges of estimations for MEs in different stages may be because of the poor quality of studies with diversity in definitions, methods, and populations. For gaining better insights into ME in Iran, we suggest studying sources, underreporting of, and preventive measures for MEs.

## Background

Medication error (ME), as a universal problem [1], is one of the most common types of medical errors [2,3]. It is also the most common single preventable cause of adverse drug events [4].

ME is defined as “any preventable event that may cause or lead to inappropriate medication use or patient harm while the medication is in the control of the health care professional, patient, or consumer.” (US National Coordinating Council for Medication Error Reporting and Prevention: NCC MERP) [5]. MEs may cause serious consequences which affect millions of patients every year [6] including death, disability, prolonged hospitalization, and also physical and psychological harm [7]. Thus, not only they can be costly, which ultimately affect the whole society [6], but also they can negatively influence patient safety [3,7]. Prevalence of MEs and its potential harm to patients make them valuable safety indicators in hospitals and healthcare system which should be reported and analyzed reliably [8].

As medication process has different stages, various estimations of error prevalence have been provided by researchers and evaluators worldwide. For example, the UK’s National Patient Safety Agency has reported that errors occur almost 50% in administration, 18% in dispensing, and 16% in prescribing stages. Moreover, the most serious incidents caused by errors happen 41% in administration and 32% in prescribing stages [9]. The US Agency for Healthcare Research and Quality (AHRQ) has reported that 20% of medical errors which lead to death or injury are from MEs [10].

It is hard to determine the actual prevalence of medication errors [8] because of the extensive variations in the reported incidence [11,12] and ME categorization [12]. Universally, lack of established definitions [12,13] along with different methods and criteria for measurement of MEs [13], affect the reliability of assessments in different settings [2,12] and countries [12] which provides incomplete image of the actual prevalence of MEs [13].

Establishing a system based on spontaneous and voluntary reporting is essential for drug safety surveillance [14]. Nevertheless, there is no established active continuous reporting system for MEs in Iran. Furthermore, shortage of consistent assessment reports is another challenge for quantifying the status of ME.

In developing countries, inadequate studies on ME, shortage of high-quality studies with well-designed methodologies in addition to wide variations in reported ME, add to difficulties in providing reliable evaluations for MEs [1]. Iran, like other developing countries, is vulnerable to unreliable assessments for MEs [3].

Even though, we know that some studies have been conducted with abovementioned limitations and challenges in our country, but in order to clarify the incidence and types of MEs thoroughly, we aimed to identify and review current available published studies on this subject.

## Methods

### Databases

In order to review Persian and English language-literature on medication errors in Iran, we searched these English electronic databases to find articles related to errors in prescription, transcription, dispensing, and administration: Scopus, Web of Science, PubMed, the Cumulative Index to Nursing & Allied Health Literature (CINAHL), and EBSCOHOST. We searched these Persian electronic databases: IranMedex, and Scientific Information Database (SID). We also manually searched references within articles to identify additional original papers. The time span was up to October 2012.

### Search terms

We used these English terms and their corresponding Persian equivalents: administration error(s), administration mistake(s), dispensing error(s), dispensing mistake(s), documentation error(s), drug mistake(s), medication error(s), medication mistake(s), nurse(s), pharmacist(s), physician(s), prescribing error(s), prescribing mistake(s), transcribing error(s), transcribing mistake(s), wrong calculation(s), wrong dose(s), wrong drug(s), wrong medication(s), and wrong route(s) of administration. Each of the words were combined using “OR” and then combined using “AND” with (Iran OR Iranian OR I.R.Iran).

### Inclusion/exclusion criteria

We considered all types of original studies on adults and children; i.e., clinical trials, longitudinal, cohort, case–control, and cross-sectional studies. We looked for studies which reported types, definitions and severity of MEs. Letters, case reports, conference papers, organizational reports, opinions or editorial papers were excluded. We also excluded articles focused on medical (not medication) errors and nursing practice errors. Moreover, we eliminated articles on preventive measures which were solely focused on usability and acceptability of measures, not on the outcome of ME.

### Selection, reading and information extraction

Two authors independently selected and one of them reviewed the articles by following these stages:

Inclusion and exclusion criteria were assessed both in reading the titles and abstracts of our search results.

The data extraction tables were completed for each article using these characteristics: types; unit of observation; sample size; study design and/or measurement tool; reported outcomes; main findings; language; ME definitions; specific types of ME definition; specialty of data collectors and corresponding authors. Then we found all full-texts of the articles selected and the exclusion criteria were also applied to the full-texts.

We classified the results from studies on types of MEs using “types of drug errors per stage of the drug delivery process” proposed by Allard *et al*. in 2002 [15]. Allard *et al*. has proposed prescribing, transcribing, dispensing and administration stages in the drug delivery process.

We considered the results reported by more than 30% of studies in every category as the most frequent topics. We report the error prevalence in different units of observations based on their most frequent percentages.

## Results

### Search results

**Figure 1:**
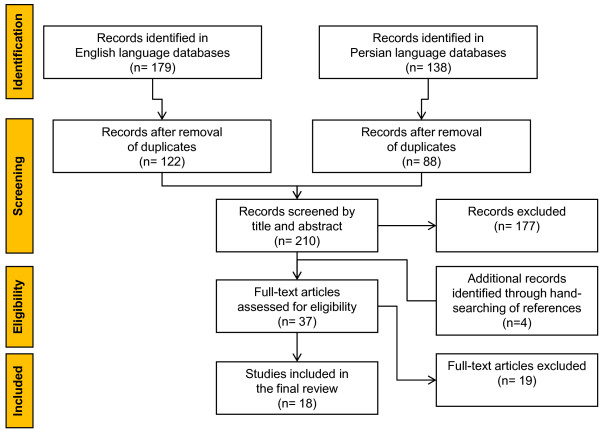
Search process and number of eligible studies.

Initially, 122 and 88 studies were identified in English and Persian biomedical databases respectively, after removing the duplicates. Of the 210 studies, 177 were of no relevance to the current review according to their titles and abstracts. Seven studies did not meet the inclusion criteria according to their full-text. After hand-searching of the reference lists of all primary studies, we added another 4 studies; this left us with 30 eligible studies for review. Out of 30 studies, 18 assessed types of medication errors. Figure 1 summarizes the complete process of selection.

### Characteristics of included studies

The time span of studies was from 2006 onwards. Other characteristics of studies on medication error included in our final review with their most frequent topics covered are summarized in Table 1.

**Table 1:** Characteristics of studies on types of MEs with their most frequent topics covered

**# of studies**	**Publication years**	Units of observation (# of studies) *	**Study designs**	Most frequent topics covered (# of studies) **
				**Administration**: **15**
				Wrong dose: 9
				Wrong infusion rate: 7
				Wrong medication: 5
		Nursing Students: 5		Wrong time: 5
		Nursing staff: 5		**Prescribing**: **6**
		Patients’ medical charts: 4		Wrong dose: 5
18	2006–2012	Dose administration by nurses: 2	Cross-sectional	Wrong medication:4
		Infusion Pumps: 1		Wrong rout : 2
		Midwives: 1		Wrong frequency: 2
				**Dispensing**: **5**
				Wrong dose: 3
				Inappropriate diluents: 2
				**Transcribing**: **3**
				Unauthorized medication: 2

* A few studies included more than one units of observation. So, the total units of observations may surpass the total number of studies.

** Topics which had been covered in over 30% of studies were mentioned as most frequent.

Eleven studies (61.1%) have been written in Persian and only 7 (38.9%) were in English. All study designs were cross-sectional and conducted in hospital settings. Nearly half of the studies (8 out of 18) used questionnaires for data gathering whereas 5 out of 18 studies used direct observation. Nursing staff and nursing students were the most frequent populations under observation (12 studies; 66.7%). The most common population introduced as corresponding authors was from the nursing staff (10 studies; 55.5%) followed by clinical pharmacists (6; 38.9%). Detailed characteristics of studies on types of medication about their definitions, data collectors and corresponding authors are presented in Table 2.

**Table 2:** **Detailed characteristics of studies on types of MEs**’ **definitions**, **data collectors and corresponding authors**

	**Author(s)/Year**	**Language**	**ME definitions**	**Specific types of ME definition**	**Data collectors**	**Corresponding author**
1	[16] Mousavi M, *et al*. 2012	E	Modified	Prescribing error def	Clinical pharmacist	Clinical pharmacist
2	[17] Vazin A, *et al*. 2012	E	NCCMERP	NA	Pharmacist	Clinical pharmacist
3	[18] Cheraghi MA, *et al*. 2012	P	NA	NA	NA	Nursing residency student
4	[19] Ebrahimi Rigi Tanha Z, *et al*. 2012	P	NA	NA	NA	Nursing group
5	[20] Khalili H, *et al*. 2011	E	NCCMERP	Administration and prescribing error def.	Clinical pharmacist	Clinical pharmacist
6	[21] Zahmatkeshan N, *et al*. 2010	P	NA	NA	NA	Nursing group
7	[22] Vessal G. 2010	E	ASHP	Prescribing error def.	Clinical pharmacist	Clinical pharmacist
8	[23] Mohammadnejad E, *et al*. 2010	P	Modified	NA	NA	MS in nursing
9	[24] Mohsenzade A, *et al*. 2010	P	NCCMERP	NA	NA	MD, Associate professor
10	[25] Fahimi F, *et al*. 2009	E	NCCMERP	Transcribing error def.	NA	Clinical pharmacist
11	[26] Nikpeyma N, *et al*. 2009	P	NCCMERP	NA	NA	MS in nursing
12	[27] Baghcheghi N, *et al*. 2008	P	NA	NA	MS in nursing	MS in nursing
13	[28] Fahimi F, *et al*. 2008	E	NCCMERP	NA	Pharmacist	Clinical pharmacist
14	[29] Koohestani HR, *et al*. 2008	P	NA	NA	NA	Senior lecturer in nursing (instructor)
15	[30] Koohestani HR, *et al*. 2008	P	NA	NA	NA	MS in nursing
16	[31] Vallizade F, *et al*. 2008	P	NA	NCCMERP	NA	Instructor of Pediatric Nursing
17	[32] Fahimi F, *et al*. 2007	E	NCCMERP	Prescribing and Transcribing error def.	clinical pharmacy resident	Clinical pharmacist
18	[33] Penjvini S, *et al*. 2006	P	NA	NA	NA	Nursing instructor

E: English, P: Persian, NA: not available, def: definition.

Many studies reported frequencies for ME types separately for different categories (prescribing, transcribing, dispensing, and administration) and most of them did not report the overall frequency of MEs aside from ME types. All studies reported the frequency of each type of MEs as percentages (Table 3).

**Table 3:** Detailed characteristics of studies on types of medication error with their most frequent findings

	**Author(s)/Year**	**Unit of observation**	**Sample size**	**Study design/Detecting method**	**Types of medication errors (in descending order)**	**Findings**
1	[16] Mousavi M, *et al*. 2012	Patients	450	Cross-sectional; Medical record	**Prescribing**	
					− Wrong rate [of IV fluid administration]	29.8%
					− Wrong dose [volume of fluid]	26.5%
					− Wrong medication [type of fluid]	24.6%
2	[17] Vazin A, *et al*. 2012	Administered doses by nurses	307	Cross-sectional; Direct Observation	**Prescribing**	**35**.**1**%
					− Monitoring §	9.5%
					− Wrong medication	7.4%
					− Wrong dosage form	6.8%
					− Wrong dose	5.9%
					**Administration**	**61**.**0**%
					− Wrong technique §	20.4%
					− Wrong time	10.0%
					− Wrong preparation	10.0%
					− Wrong dose	7.7%
3	[18] Cheraghi MA, *et al*. 2012	Nurses	64	Cross-sectional; self report survey (Questionnaire)	**Administration**	
					− Wrong [infusion] rate	44.7%
					− Wrong dose	23.4%
4	[19] Ebrahimi Rigi Tanha Z, *et al*. 2012	Nursing students	54	Cross-sectional; self report survey (Questionnaire)	**Dispensing and**/**or preparation**	
					− Wrong concentration	33.4%
					− Wrong volume	25.9%
					− Wrong dose	22.2%
					**Administration**	
					− Wrong time	20.6%
					− Failing to check [oral] medication- food interaction	14.7%
					− Omission	11.4%
5	[20] Khalili H, *et al*. 2011	Medical charts	861	Cross-sectional; Chart review	**Prescribing**	
					− Wrong dose	38.4%
					− Wrong medication	33.0%
					**Administration**	
					− Omission [Medication not taken/administered at all]	14.3%
					− Wrong medication	5.4%
6	[21] Zahmatkeshan N, *et al*. 2010	Nursing staff; Midwives	332	Cross-sectional; self report survey (Questionnaire)	**Administration**	
			68		− Wrong dose	37.7%
					− Wrong medication	27.7%
					− Wrong route	18.3%
7	[22] Vessal G. 2010	Patients	818	Cross-sectional; Chart review	**Prescribing**	
					− Wrong frequency	37.2%
					− Wrong medication	19.8%
					− Wrong dose	16.3%
8	[23] Mohammadnejad E, *et al*. 2010	Nursing students	78	Cross-sectional; self report survey (Questionnaire)	**Administration**	
					− Wrong dose	24.3%
					− Wrong medication	18.9%
					− Wrong [infusion] rate	16.2%
9	[24] Mohsenzade A, *et al*. 2010	Pediatrics’ medical charts	2250	Cross-sectional; Medical records	**Prescribing**	**46**.**3**%
					− Wrong dose	37.0%
					− Wrong frequency	28.0%
					− Wrong route	19.0%
					**Transcribing**	**10**.**0**%
					**Dispensing**	**11**.**3**%
					**Administration**	**32**.**4**%
10	[25] Fahimi F, *et al*. 2009	Pediatrics’ medical charts	287	Cross-sectional; Direct observation	**Transcription**	**51**.**8**%
					− Omission	52.0%
					− Wrong dose	18.0%
					− Unauthorized medication*	16.0%
					− Replacing medication without physician’s approval	7.0%
					− Requesting more than required according to order	7%
11	[26] Nikpeyma N, *et al*. 2009	Nurses	100	Cross-sectional; self report survey (Questionnaire)	**Administration**	
					− Wrong dose	27%
					− Omission	22%
					− Wrong time	18%
12	[27] Baghcheghi N, *et al*. 2008	Nursing students	372	Cross-sectional; Direct observation	**Dispensing and**/**or preparation**	**13**.**4**%
					− Inappropriate diluents	2.7%
					− Forgetting to prepare medication	2.2%
					− Wrong dose	1.9%
					**Administration**	**27.8**%
					− Wrong [bolus] rate	11.6%
					− Wrong [IV injection] rate	9.1%
					− Wrong route [of injection]	3.2%
13	[28] Fahimi F, *et al*. 2008	IV injections administered by nurses	524	Cross-sectional; Direct observation	**Dispensing and**/**or preparation**	**33**.**6**%
					− Wrong dose	
					− Inappropriate diluents	
					− Inappropriate storage	
					**Administration**	**66**.**4** %
					− Wrong [bolus] rate	
					− Wrong [infusion] rate	
14	[29] Koohestani HR, *et al*. 2008	Nursing students	76	Cross-sectional; self report survey (Questionnaire)	**Administration**	
					− Wrong dose	22.0%
					− Wrong medication	20.3%
					− Wrong [infusion] rate	18.6%
15	[30] Koohestani HR, *et al*. 2008	Nursing students	60	Cross-sectional; self report survey (Questionnaire)	**Administration**	
					− Wrong [infusion] rate	28.6%
					− Wrong dose	17.1%
					− Wrong medication	14.3%
16	[31] Vallizade F, *et al*. 2008	Pediatrics’ medical charts	898	Cross-sectional; Medical records	**Prescribing**	
					− Not highlighting administration considerations §	74.1%
					− Wrong time	47.8%
					− Illegible or ambiguous handwriting	45.5%
					**Administration**	
					− Not pursuing administration considerations §	77.5%
					− Failing to check interactions	14.9%
					− Wrong time	14.8%
17	[32] Fahimi F, *et al*. 2007	Infusion pump doses	43	Cross-sectional; Direct observation	**Transcribing**	
					− Unauthorized medication	10%
					**Administration**	
					− Wrong dose and rate	70%
					**Dispensing**	
					− Inappropriate labeling	20.0%
18	[33] Penjvini S, *et al*. 2006	Nursing staff	104	Cross-sectional self report survey (Questionnaire)	**Administration**	
					− Omission	42.5%
					− Wrong dose	15.1%
					− Wrong time	13.7%

* Unauthorized medication: those medications that were administered but could not be found in physician’s orders.

§ Vazin and Delfani, have categorized “wrong technique” and “monitoring” under administration and prescribing respectively [17]. Vallizade *et al*. also have categorized “Not pursuing administration considerations” and “Not highlighting administration considerations” under administration and prescribing respectively [31]. These categorizations are not in accordance with our classification based on the article by Carthey [15].

IV: Intravenous; IM: Intramuscular.

### Medication error definitions

Eleven studies provided their definitions for MEs; but, they used different definitions. Eight out of 18 studies used NCC MERP definition, 2 studies used their own modified definitions and 1 of them used AHSP (American Society of Health-System Pharmacists) definition. ME definitions were not clear or defined in the remaining 7 studies.

Specific definition for types of medication errors detected was provided by only five studies (27.8%); others used general definitions or no definition. All the five studies have been done by clinical pharmacists. Of the 15 studies on administration error, only 2 (13.33%) provided specific definitions for administration errors [20,32]. Three studies out of 6 (50%) on prescribing error used specific definitions [16,20,22]. Definitions for transcribing error were provided in two out of 3 studies (66.7%) [25,32]. No specific definition was mentioned in studies on dispensing errors.

### Administration errors

Most of studies (15 of 18 studies; 83.3%) reported administration errors with reported prevalence between 14.3% and 70.0% in administration stage.

Overall estimations for administration errors based on different units of observation were as follow; 61.0% to 70.0% in administered doses, 32.4% in pediatric medical charts, 27.0% to 44.7% in nursing staff, 20.6% to 28.6% in nursing students, and finally 14.3% in adult medical charts.

In administration stage, wrong medication was reported only in nursing students (14.28-27.7%) and wrong time only in nurses (10.0- 18.0%). Wrong rate of injection was reported in three studies by nurses; only one out of these three studies reported estimation for the error prevalence (44.7%). Wrong rate of injection was reported in four studies by nursing students; all of them reported estimation for the error prevalence between 11.5% and 28.7%. In comparison, wrong dose error prevalence in administration was higher in nursing students (17.4%-37.7%) than nurses (7.7%-27%).

### Prescribing errors

Thirty three percent of studies reported prescribing errors and the error prevalence ranged from 29.8% to 47.8% in different studies.

Prescribing errors varied in different studies depending on their units of observations; 38.4% to 47.8% in medical charts, 29.8% to 37.2% in patients, and 35.1% in administered doses.

Most of the studies which reported errors in prescription stage were conducted by clinical pharmacists. Five out of 6 studies in this category used medical records or chart reviews as the error detection method. Only 1 of them used direct observation for measuring errors in prescription.

Wrong dose prevalence in prescribing stage varied from 5.9 to 37.0%, and wrong medication ranged between 7.4 and 33.0% in different studies.

The lowest prescribing error prevalence was reported in the study conducted by clinical pharmacist who used direct observation as error detection method [17]. The highest prescribing error prevalence was reported in the study conducted on pediatrics medical records, while none of the most common types of prescribing errors were reported in the last study [31].

### Dispensing errors

The prevalence of dispensing errors was assessed by 5 studies and differed from 11.3% to 33.6%. It was also variable in different units of observation; 33.6% in administered doses, 20.0% in infusion pump doses, 13.4% in nursing students and 11.3% in medical charts.

Dispensing errors were detected by direct observation (3 studies), questionnaire (1 study) and medical records (1 study).

Wrong dose error in dispensing stage ranged between 1.9% and 2.7%. The lowest prevalence of dispensing error was detected in the study which used direct observation [27].

### Transcribing errors

Transcribing errors was assessed in three studies. The prevalence of this type of error was between 10.0% and 51.8% in pediatric medical charts; another study estimated transcribing errors as 10.0% for infusion pump doses. The most frequently reported transcribing error was unauthorized medication which varied between 10.0% and 16.0% in two studies.

Two studies used direct observation and one used medical records review for detecting transcribing errors.

### Severity of MEs

Three studies reported findings on severity of MEs [16,22,32]. For categorizing MEs and determining the severity of them, 2 studies used National Coordinating Council for Medication Error Reporting and Prevention (NCC MERP) Index [16,32]; the other one used definitions and leveling provided by Hartwig, Denger, and Schneider [22].

Fahimi *et al*. assessed the severity of errors in preparation and administration of medications by infusion devices; they reported that labeling errors, unauthorized medications, and rate-concentration inconsistencies all belonged to category B [32]. Mousavi *et al*. investigated fluid therapy errors in medical wards; they found that the most common type of error was wrong rate of fluid administration (29.8%) in severity categories D (45.5%), C (44.8%), and E (7.9%) [16].

In the study on prescribing errors in nephrology wards, Vessal reported level 1 as the most common severity category (77 observed errors; 89.5% of all), in which errors had occurred but had not resulted harm to patients [22].

## Discussion

The aim of our study was to clarify the incidence and types of medication errors by reviewing current available literature on MEs in Iran. Our findings show that the most reported type of ME and highest percentage for any type of error is in the administration error stage.

### Studies characteristics

All studies in our review had been conducted after 2005. This shows that studying MEs in Iran is a relatively new area of research considering the duration of only six years for active publication. Research on ME has started since 1960 and grew steadily from 1990 onwards [34]. The first published studies included in different reviews of the literature date back to 1984–1985 [12,35]. But, in two reviews by Lewis *et al*. [35] and Lisby *et al*. [12], most included studies were done after 2000 and between 2005 and 2006 respectively.

We found 18 studies which assessed types of MEs. But, in a systematic review by Alsulami *et al*. 10 related studies included from Iran related to types of MEs [1]. This difference in the included articles may be due to the language bias because many of the articles were in Persian and had been translated [1]; in our study, only 7 out of 18 articles were in English. Moreover the quality assessment by Alsulami *et al*. was an additional value-added step in comparison to our review; this may have caused dropping off those studies with lower quality in their study.

In order to provide a comprehensive estimation of the prevalence of MEs, the ideal approach is using multiple error detection methods all together which maximizes probability of error detection in every stage of the medication process [11,35,36]. But, none of our included studies used multiple error detection methods concurrently and their estimates just relied on one (usually non-overlapping) error detection method.

Additionally, follow-up studies are required to evaluate the status of MEs in depth [7]; but we did not find any follow-up or repeated study whit the same settings in the 6 years time frame. Moreover, there is a huge shortage of interventional studies with specific focus on decreasing prevalence of medication errors. Recently, protocols of randomized controlled trials have been published to assess the effectiveness of audit and feedback on physician prescribing [37].

### Medication error definitions

We found that, ME definitions were different from one study to another [12,36]. Most studies (45%) used the NCC MERP general definition for ME ; this figure is close to 38% reported in the systematic review by Lisby *et al*. [12]. Nearly 50% of our studies which reported prescribing errors used specific definitions in comparison to 38% of studies included in the review of Middle Eastern studies by Alsulami *et al*. [1]. Just above 13% of our studies used specific definitions for administration errors in comparison to 27% reported in the systematic review by Alsulami *et al*. in the Middle East [1]. In order for error prevalence to be comparable, the definitions used in different studies should be as similar as possible [38]. But, regarding the fact that there is no standard definition available for ME [38], inconsistencies in ME assessment inevitably occur. This is in agreement with the findings reported by Lisby *et al*. [12].

### Overall typology of ME

Wrong dose or error in dosage was the most commonly reported type of errors regardless of medication process. This finding is in accordance with many other studies as well [1,12,15,35,39,40]. It seems that the prevalence of wrong dose errors in our study (2% to 38%) -although wide- is pretty close to the Middle Eastern countries (0.15% -35%) [1].

In Iranian studies the most reported types of MEs were wrong dose followed by wrong intravenous (IV) administration rate (infusion or bolus), wrong medication, wrong time and omission. These types can be found in other reviews as the most frequent errors [1,10,15,35,36,40,41], except for the wrong rate of IV injections.

### Administration errors

The most frequent reported type of ME in our studies was administration error. This is in contrast to other reviews that stated the majority of MEs have occurred in the prescribing stage [1,34,36]. This finding might have been because most of our studies had been performed by nursing groups involved in administration -not prescribing - of medications.

Regardless of the unit of observation in our studies, administration errors had highest percentage of prevalence in all stages of medication process followed by prescribing errors. Error prevalence in administration stage in our study (14.3%-70%) is somehow close to the other Middle Eastern countries (9.4%- 80%) [1]. Administration errors, although with lower prevalence than ours, has also been the most common type of MEs in developed countries (47%) [10].

Most studies which reported administration errors used nurse-completed questionnaires as their only data gathering tool. But, using questionnaires is not recommended as the main data gathering tool to evaluate types of ME [10,12,35,36]. The main reason is that it cannot determine the prevalence of MEs due to the lack of valid denominators [12] or inconsistencies in using reliable denominators [36]. It has been suggested to consider using questionnaires as an additional tool along with other data gathering methods [35]. In contrast, direct observation, as the second most popular data gathering method in our studies, is considered to be the most sensitive way of detecting administration errors [12].

Medical record or chart reviews were used infrequently in Iranian studies, some authors have stated that these tools can lead to underestimation of administration errors [12,35].

### Prescribing errors

Prescribing errors were the second most common type of ME in our studies. This is not in accordance to many other studies which accounted prescribing errors as their first most frequent type of ME [10,11,15,35,36]. The upper limit of prescribing errors prevalence in our study (29.7%-47.8%) is much lower than the reported figures in the Middle Eastern countries (7.1%-90.5%) and higher than reported by high-income countries like UK (2-14%) [1]. Nevertheless, inappropriate physician prescribing has been shown to provide major concerns in clinical settings [37].

Lewis *et al*., which included only studies on prescribing errors, showed that the majority of data collectors were pharmacists [35]. Detection of prescribing errors is usually done by pharmacists or clinical pharmacists, especially in the hospital settings [16,20,22]. But, currently there are just few practicing clinical pharmacists in Iranian hospitals and medical wards [20]. This can be one of the future orientations for research and development in ME management.

In Iranian studies, three data gathering method were used for detecting prescribing errors; medical records review, chart review and direct observation. Based on the evidence, chart review is the most appropriate method for detecting of prescribing errors [35,36]. Medical records review has higher rates of error detection, but it is prone to biases imposed from incomplete documentation [35].

### Dispensing errors

The prevalence of dispensing errors in our studies varied between 11.3% and 33.6%; this is higher than the reported figures in other studies [15,42]. But, in one systematic review on dispensing errors in pediatric wards, the estimated prevalence varied between 5% and 58% [36], which is much wider than situation in Iran. We encountered the shortage of studies that provided information about this stage of the medication process, both in our studies and also in other reviews.

### Transcribing errors

The prevalence of transcribing errors in our studies varied between 10.0% and 51.8%; this is also higher than the reported figures in other studies [15,42]. Alsulami *et al*. included just one study on transcribing errors [1], which we reviewed the same study as well. The shortage of studies on this stage of medication process is a major challenge in providing precise estimation of transcribing errors.

### Severity of MEs

We found only three studies that reported severity of MEs and Alsulami *et al*. included only one [1]. This shows the shortage of evidence on outcomes of MEs in Iran, and accordingly the serious lack of monitoring and analyzing the adverse outcome of these errors in Iran.

## Conclusion

The aim of this review was to evaluate the status of MEs in Iran according to available published evidence. We encountered wide ranges of estimations for MEs in different stages mostly because of the poor quality of studies with diversity in definitions, methods, and populations. Although, the comparison of ME estimations is difficult, the reported prevalence of errors is higher than other countries.

At last, for gaining a more comprehensive insight into ME in Iran, we propose studying sources, underreporting of, and preventive measures for MEs.

## Competing interests

The authors report no conflicts of interest. The authors alone are responsible for the content and writing of the paper.

## Authors’ contributions

MA, drafted the manuscript, and participated in acquisition of data and data analysis. AA, participated in drafting the manuscript and Data analysis. HM, revised manuscript critically and has given final approval of version to publish. KM, participated in drafting the manuscript. JM, participated in Acquisition of data. GK, participated in designing the study and revised manuscript critically. All authors read and approved the final manuscript.
